# Mitochondria and Endoplasmic Reticulum in Diabetes and Its Complications

**DOI:** 10.1155/2012/985075

**Published:** 2012-03-13

**Authors:** Ki-Up Lee, Robert A. Harris

**Affiliations:** ^1^Department of Internal Medicine, College of Medicine, University of Ulsan, Seoul 138-736, Republic of Korea; ^2^Department of Biochemistry and Molecular Biology, School of Medicine, Indiana University, Indianapolis, IN 46202-5122, USA

Recent studies have provided evidence that mitochondrial dysfunction and endoplasmic reticulum (ER) stress are major pathogenic factors for diabetes and its complications. Mitochondria are the most important source of the chemical energy required by cells. Regulation of the role that mitochondria play in the metabolism of glucose and fatty acids, the primary fuels used by cells to produce ATP, has been the subject of intensive research for several decades. In spite of this, much remains to be learned about tissue-specific fuel selection and the pathogenesis of diabetes and its complications. Mitochondria also play an important role in the generation of reactive oxygen species (ROS) and cell apoptosis. The ER is a major subcellular compartment involved in calcium homeostasis, lipid synthesis, and protein folding and maturation. Various factors that interfere with ER function lead to accumulation of unfolded proteins. This triggers downstream signaling pathways, which is called the unfolded protein response (UPR). Although this is an adaptive mechanism to resolve ER stress, chronic UPR activation may lead to cell injury. Cellular homeostasis also depends upon the functional relationship between mitochondria and the ER. Propagation of calcium signaling from ER to mitochondria is involved in both ATP production and cell death. On the other hand, the ER requires ATP to function properly, which may make it the best site for sensing metabolic stress.

In this special issue of the journal we have assembled several invited reviews, from well-recognized investigators, on the roles of mitochondrial dysfunction and ER stress in the pathogenesis of diabetes and its complications. Some papers also deal with important issues like mitochondrial biogenesis, mitochondrial fusion/fission, and autophagy in the diabetic state.

Dr. A. Naudi et al. reviewed the mechanism of cellular dysfunction in response to mitochondrial oxidative stress. Increases in plasma glucose and free fatty acid (FFA) cause mitochondrial overproduction of ROS. This leads to several maladaptive responses including blockade of glycolysis and accumulation of upstream glycolytic metabolites, PARP activation and consequent increases in the production of inflammatory mediators, and protein oxidative damage. They also suggested the use of antioxidants, uncouplers, or PARP inhibitors for the prevention or reversal of diabetic complications.

Dr. Z. A. Ma et al. discussed the molecular mechanism of mitochondrial dysfunction-induced cell injury. In pancreatic beta cells, mitochondrial ROS produced by metabolic stress activates UCP2, which leads to proton leak across the mitochondrial inner membrane. This reduces beta cell synthesis of ATP and reduces glucose-stimulated insulin secretion. In addition, ROS oxidizes polyunsaturated fatty acids in mitochondrial membrane phospholipids (cardiolipin), and this impairs membrane integrity and leads to cytochrome c release into the cytosol and apoptosis. Group VIA phospholipase A_2_ (iPLA_2_beta) appears to provide a mechanism for repairing mitochondrial phospholipid damage. The authors suggested that interventions that attenuate the adverse effects of ROS on beta-cell mitochondrial phospholipids may represent a means for preventing the development of type 2 diabetes.

Dr. B. Ponugoti et al. reviewed the role of the FOXO family of forkhead transcription factors in the regulation of cellular oxidative stress response pathways. FOXO proteins are known to play an important role in protection of cells against oxidative stress. However, in response to certain ROS levels, FOXO proteins switch from prosurvival to proapoptotic signaling, resulting in cell death. In the diabetic state, the induction of FOXO by hyperglycemia plays an important role in the generation of proinflammatory cytokines. On the other hand, insulin signaling inactivates FOXO1. The authors suggested that activated FOXO1 disrupts the mitochondrial electron transport chain, negatively affecting fatty acid oxidation. 

Dr. A.-M. Joseph et al. reviewed skeletal muscle mitochondrial metabolism with special emphasis on mitochondrial biogenesis, mitochondrial fusion/fission, and autophagy. Mitochondrial biogenesis is induced by numerous physiological, environmental, and pharmacological stimuli and is regulated by various factors including PGC-1, NRF 1/2, and SIRT1–7. In the diabetic state, these processes become deregulated and the ability of the cell to respond to environmental changes is diminished. The potential to stimulate mitochondrial biogenesis through physiological interventions such as exercise, caloric restriction, or pharmacological mimetics of mitochondrial biogenesis can be promising in improving insulin sensitivity. The paper also described mitochondrial dynamics (fusion/fission) and autophagy. Levels of the fusion proteins Mfn2 and OPA1 are reduced in skeletal muscles of diabetic patients, suggesting mitochondrial fusion is an important signaling event for mitochondrial biogenesis in muscle. 

Dr. S. H. Back et al., Dr. U. Karunakaran et al., Dr. B. Basha et al., and Dr. J. Xu et al. separately reviewed the roles of ER stress in the pathogenesis of beta-cell failure, endothelial dysfunction, and diabetic cardiomyopathy, respectively. ER plays a central role in protein folding and in quality control of newly synthesized proteins. The ER also serves as an essential site for synthesis of lipids and for high-capacity buffering of intracellular calcium. Increased demand or decreased ability to fold proteins in the ER leads to accumulation of unfolded proteins in the ER, a state called “ER stress.” In this state, a series of compensatory mechanisms are induced, collectively termed UPR, that include inhibition of protein translation, increased expression of ER chaperones, ER-associated degradation, and cell apoptosis. Although this is an adaptive mechanism to resolve ER stress, chronic UPR activation may lead to cell injury. In the diabetic state, high plasma glucose and FFA levels are well known to cause cellular dysfunction and injury, which is called “glucotoxicity,” “lipotoxicity,” and “glucolipotoxicity” when the two are combined. These metabolic stresses are known to induce ER stress and UPR.

Dr. Y. Tanaka et al. reviewed the role of autophagy in the pathogenesis of diabetic nephropathy. Autophagy is a major catabolic pathway involved in degrading and recycling macromolecules and damaged organelles to maintain intracellular homeostasis. This dynamic process involves membrane formation and fusion and includes autophagosome formation, autophagosome-lysosome fusion, and the degradation of intra-autophagosomal contents by lysosomal hydrolases. The authors summarized recent studies showing that autophagy in podocytes and renal tubular epithelial cells plays a renoprotective function under several pathologic conditions.

Finally, Dr. J. Leem and Dr. E. H. Koh reviewed the functional relationship between mitochondria and the ER. Recent studies using electron tomography demonstrated that the outer mitochondrial membrane and the ER are joined by tethers, enabling ER proteins to associate directly with proteins and lipids of the outer mitochondrial membrane. Many papers to date have shown that ER stress induces mitochondrial dysfunction to cause cell apoptosis. In addition, recent lines of evidence suggest that the reverse is also happening; mitochondrial dysfunction induces ER stress to decrease adiponectin synthesis in adipocytes and to cause hepatic insulin resistance. Thus, it appears that bidirectional communications exist between these two organelles. Future studies are needed to carefully dissect the interactions between mitochondria and ER.

**Ki-Up Lee** **Ki-Up Lee** **Robert A. Harris** **Robert A. Harris**

